# Prognostic value of the serum creatinine/albumin ratio for 28-day mortality in heart failure: a retrospective cohort study

**DOI:** 10.3389/fcvm.2025.1586327

**Published:** 2025-07-09

**Authors:** Hao Luo, Xinqi Chen

**Affiliations:** ^1^ Department of Cardiology, Xiangan Hospital Affiliated to Xiamen University, Xiamen, Fujian, China; ^2^ Department of Gastroenterology, First Hospital of Quanzhou Affiliated to Fujian Medical University, Quanzhou, Fujian, China

**Keywords:** heart failure, creatinine/albumin ratio, mortality, retrospective cohort study, biomarker

## Abstract

**Background:**

Heart failure (HF) is a global health challenge with high morbidity and mortality. The serum creatinine/albumin ratio (CAR), a marker of renal dysfunction and malnutrition, has shown prognostic value in other critical illnesses but remains underexplored in HF patients.

**Methods:**

This retrospective cohort study included 1,893 HF patients hospitalized at the Fourth People's Hospital of Zigong, China, between December 2016 and June 2019. Cox proportional hazards models assessed the association between CAR and 28-day mortality. Dose-response relationship was assessed using restricted cubic spline analysis, Kaplan–Meier curves illustrated survival differences, and Receiver Operating Characteristic (ROC) analysis evaluated CAR's predictive performance.

**Results:**

Patients with CAR ≥ 3.5 were older, had worse cardiac function, and had more comorbidities than those with CAR < 3.5.A linear relationship was observed between CAR and 28-day mortality. Each 1-unit increase in CAR was associated with a 14% higher mortality risk (HR: 1.14, 95% CI: 1.07–1.21, *p* < 0.001). ROC analysis showed that CAR had an AUC of 77.1%, which was slightly higher than creatinine alone (76.2%) and markedly better than BNP (68.0%) and albumin alone (64.9%).

**Conclusion:**

In patients with HF, CAR may serve as an independent predictor of 28-day mortality. Its ability to simultaneously reflect renal dysfunction, malnutrition, and inflammation highlights its potential as a valuable biomarker for risk stratification. Further multicenter, prospective studies are needed to confirm its clinical utility and investigate its role alongside other biomarkers in guiding personalized treatment strategies and improving patient outcomes.

## 1 Introduction

Heart failure (HF) remains a significant global health concern, with an estimated 56.19 million cases reported worldwide by 2019 (1). Its prevalence increases markedly with age, affecting less than 1% of individuals under 55 years of age, but surpassing 10% in those aged 70 years and older (2, 3). Despite advances in treatment, HF remains associated with poor outcomes, including high mortality and frequent hospital readmissions (4–6). As populations age, the burden of HF is expected to intensify, making early identification of high-risk patients critical for optimizing treatment and improving prognosis.

The serum creatinine/albumin ratio (CAR) is an emerging biomarker that integrates two routinely measured clinical parameters—serum creatinine and albumin. It reflects both renal dysfunction and malnutrition, two interrelated conditions that are highly prevalent in HF and independently associated with adverse outcomes (7–10). Creatinine is a well-established indicator of renal function, and elevated levels often reflect impaired perfusion or chronic kidney disease in HF patients (9, 11). Albumin, on the other hand, is a marker of nutritional status and systemic inflammation. Hypoalbuminemia in HF has been linked to chronic inflammation and poor survival (10, 12).

Recent studies have demonstrated the prognostic value of CAR in several critical illnesses. For example, elevated CAR has been associated with increased mortality in patients with acute coronary syndrome (7), acute pancreatitis (13), and severe burn injuries (14). These findings suggest that CAR may serve as a composite biomarker that reflects multiple pathophysiological pathways—renal impairment, malnutrition, and inflammation—that contribute to poor outcomes.

However, the role of CAR in the context of heart failure remains underexplored. Given that HF commonly involves a combination of renal dysfunction, systemic inflammation, and nutritional decline, we hypothesize that CAR may be a useful predictor of short-term mortality in this population. In this study, we aim to investigate the association between CAR and 28-day mortality in Chinese patients hospitalized for HF, with the goal of providing a simple and effective risk stratification tool to inform early clinical decision-making.

## 2 Materials and methods

### 2.1 Study population

**Figure 1:**
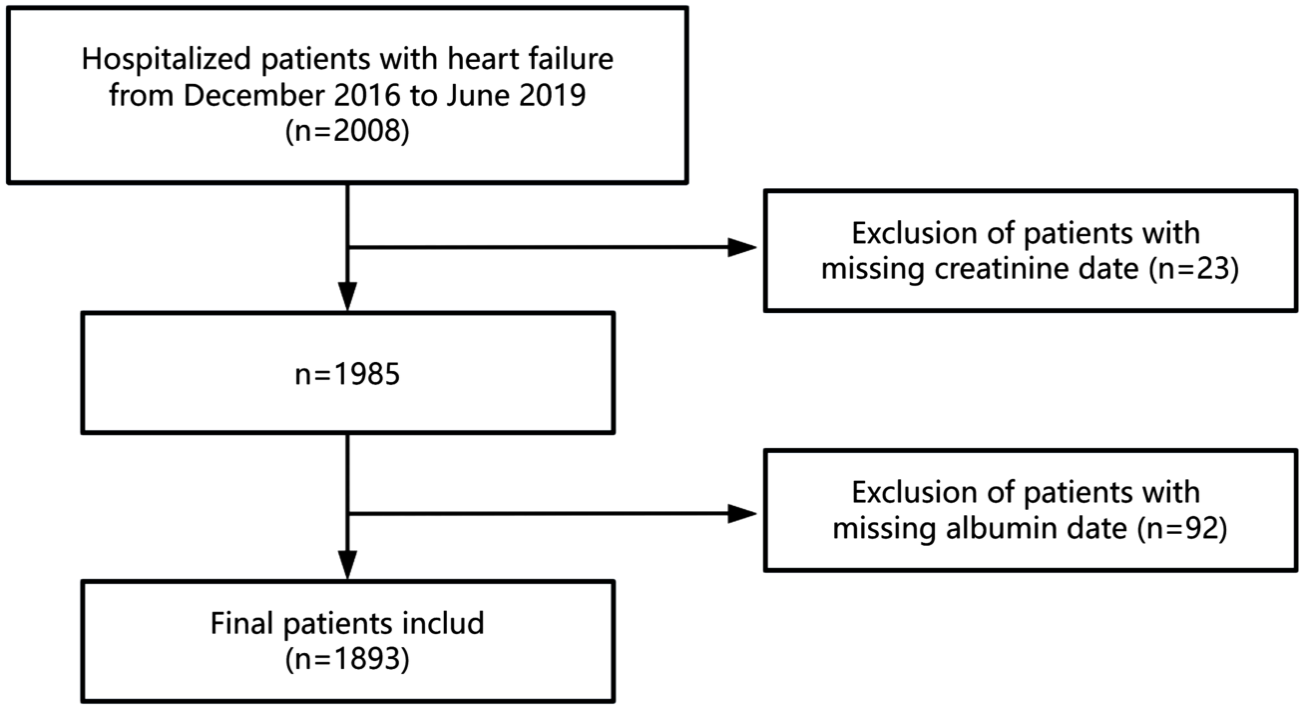
The flowchart of the participants selection.

This retrospective cohort study analyzed data from patients hospitalized with HF at the Fourth People's Hospital of Zigong, Sichuan, China, between December 2016 and June 2019. The dataset, which undergoes regular updates, is maintained by the Massachusetts Institute of Technology (Cambridge, MA, USA; https://physionet.org/content/heart-failure-zigong/1.3/) (15). The collected data encompassed demographic information, baseline clinical indicators, comorbidities, laboratory findings, therapeutic interventions, and patient outcomes. HF was diagnosed according to the criteria established by the European Society of Cardiology (ESC) (16). Patients were included if they were hospitalized with a primary diagnosis of HF. The exclusion criteria were as follows: (1) patients with incomplete serum creatinine data and (2) those with incomplete serum albumin data. Ultimately, 1893 patients met the inclusion criteria for this study. Figure 1 provides a flowchart illustrating the selection of participants. The study protocol received approval from the ethics committee of Zigong Fourth People's Hospital (Approval Number 2020-010), with informed consent waived due to the retrospective study design. All procedures conformed to the principles outlined in the Declaration of Helsinki and adhered to the STROBE (Strengthening the Reporting of Observational Studies in Epidemiology) guidelines (17).

### 2.2 Data collection

At admission, demographic data and clinical characteristics were comprehensively recorded. These included patient-specific factors such as age, gender, body mass index (BMI), mean arterial pressure (MAP), and the classification of cardiac function based on the New York Heart Association (NYHA) criteria. Additionally, comorbidities were evaluated, including histories of myocardial infarction, cerebrovascular disease, chronic obstructive pulmonary disease (COPD), diabetes, and the presence of solid tumors. Laboratory investigations conducted within the initial 24 h post-admission included measurements of white blood cell (WBC) count, hemoglobin levels, platelet count, high-sensitivity cardiac troponin (hs-cTn), brain natriuretic peptide (BNP), potassium, sodium, serum creatinine, and albumin. Our data had missing values as follows: hs-cTn had a 2.9% missing rate (55 cases), BNP had a 1.2% missing rate (22 cases), hemoglobin had a 0.9% missing rate (17 cases), WBC and platelet count each had a 0.8% missing rate (16 cases), potassium and sodium each had a 0.2% missing rate (3 cases) (Supplementary Table S1). Missing data were imputed using a multivariate imputation method. To assess the robustness of our approach, we conducted a sensitivity analysis using complete-case data only, and the hazard ratios for CAR remained consistent in magnitude and direction across all models (Supplementary Table S2), supporting the reliability of our imputation-based results.

### 2.3 Measurement of CAR

The CAR was calculated as follows: CAR = serum creatinine (μmol/L)/albumin (g/L).

### 2.4 Statistical analysis

Continuous variables were expressed as mean ± standard deviation (SD) for normally distributed data or as median with interquartile range (IQR) for skewed distributions; categorical variables were presented as percentages. Group comparisons were performed using one-way analysis of variance (ANOVA) for continuous variables with a normal distribution and the *χ*^2^ test for categorical variables. For continuous variables not normally distributed, the Kruskal–Wallis H test was employed.

The association between CAR and 28-day mortality was assessed using Cox proportional hazards regression models with progressive adjustment: Model 1 was unadjusted; Model 2 adjusted for age and gender; Model 3 further included NYHA cardiac function classification, platelet, BNP, and potassium. Covariates included in these models were selected based on their clinical significance and their potential to alter effect estimates by at least 10% (18).

The dose-response relationship between CAR and 28-day mortality was examined using restricted cubic spline analysis. Kaplan–Meier survival curves were generated to visualize cumulative 28-day mortality across CAR groups. Subgroup analyses were conducted to assess potential effect modification by age, gender, NYHA classification, myocardial infarction, and cerebrovascular disease. ROC analysis was used to evaluate the predictive performance of CAR, serum creatinine, albumin and BNP, with sensitivity, specificity, and area under the curve (AUC) reported. The optimal CAR cut-off was determined using the Youden index, and patients were categorized into two groups: CAR <3.5 and CAR ≥3.5. All analyses were performed using R software (version 3.3.2; http://www.R-project.org) and Free Statistics software (version 1.9). A *p*-value <0.05 was considered statistically significant.

## 3 Results

### 3.1 Baseline characteristics of study population

The baseline characteristics of the patients are summarized in Table 1. A total of 1,893 patients were included in the analysis, categorized into two groups based on the CAR: CAR < 3.5 (*n* = 1,429) and CAR ≥ 3.5 (*n* = 464). Patients with CAR ≥ 3.5 were generally older (≥70 years: 85.6% vs. 68.9%, *p* < 0.001), and a greater proportion were male (54.5% vs. 37.8%, *p* < 0.001). Patients with higher CAR (≥3.5) had significantly lower mean arterial pressure, worse NYHA classification, and higher prevalence of myocardial infarction and diabetes compared to those with CAR < 3.5. Several laboratory parameters showed significant differences, with the CAR ≥ 3.5 group having higher WBC, BNP, and serum creatinine levels, but lower albumin levels, compared to the CAR < 3.5 group (all *p* < 0.05).

**Table 1:** Baseline characteristics of heart failure patients.

Covariates	Total	CAR < 3.5	CAR ≥ 3.5	*P*-value
	(*n* = 1,893)	(*n* = 1,429)	(*n* = 464)	
Age, years				<0.001
<70	511 (27.0)	444 (31.1)	67 (14.4)	
≥70	1,382 (73.0)	985 (68.9)	397 (85.6)	
Gender				<0.001
Male	793 (41.9)	540 (37.8)	253 (54.5)	
Female	1,100 (58.1)	889 (62.2)	211 (45.5)	
BMI, kg/m^2^	20.8 (18.5, 23.4)	20.8 (18.4, 23.4)	20.7 (18.6, 23.4)	0.845
MAP, mmHg	94.9 ± 16.2	95.7 ± 15.8	92.4 ± 17.2	<0.001
NYHA cardiac function classification, *n* (%)				<0.001
II	331 (17.5)	262 (18.3)	69 (14.9)	
III	983 (51.9)	768 (53.7)	215 (46.3)	
IV	579 (30.6)	399 (27.9)	180 (38.8)	
Myocardial infarction, *n* (%)	136 (7.2)	93 (6.5)	43 (9.3)	0.046
Cerebrovascular disease, *n* (%)	142 (7.5)	110 (7.7)	32 (6.9)	0.569
COPD, *n* (%)	224 (11.8)	176 (12.3)	48 (10.3)	0.253
Diabetes, *n* (%)	439 (23.2)	307 (21.5)	132 (28.4)	0.002
Solid tumor, *n* (%)	36 (1.9)	25 (1.7)	11 (2.4)	0.395
WBC, 10^9^/L	7.3 ± 3.5	7.2 ± 3.2	7.8 ± 4.2	<0.001
Hemoglobin, g/L	115.0 ± 24.3	119.5 ± 22.3	101.1 ± 25.0	<0.001
Platelet, 10^9^/L	144.3 ± 64.0	143.8 ± 61.6	146.0 ± 71.0	0.508
Hs-cTn, pg/ml	0.1 (0.0, 0.1)	0.0 (0.0, 0.1)	0.1 (0.0, 0.2)	<0.001
BNP, pg/ml	774.8 (319.4, 1,764.1)	702.4 (271.8, 1,578.6)	1,108.1 (461.1, 2,528.1)	<0.001
Potassium, mmol/L	4.0 ± 0.7	3.9 ± 0.6	4.4 ± 0.8	<0.001
Sodium, mmol/L	138.3 ± 4.9	138.8 ± 4.7	136.7 ± 5.0	<0.001
Serum creatinine, μmol/L	87.4 (65.0, 123.7)	75.3 (61.2, 94.4)	167.0 (140.0, 220.1)	<0.001
Albumin, g/L	36.5 ± 5.0	37.4 ± 4.7	34.0 ± 4.9	<0.001
CAR	2.4 (1.8, 3.5)	2.0 (1.6, 2.7)	4.8 (4.0, 6.7)	<0.001
Mortality, *n* (%)	32 (1.7)	9 (0.6)	23 (5.0)	<0.001

BMI, body mass index; MAP, mean arterial pressure; NYHA, New York Heart Association; COPD, chronic obstructive pulmonary disease; WBC, white blood cell; hs-cTn, high sensitivity cardiac troponin; BNP, brain natriuretic peptide; CAR, creatinine/albumin ratio.

### 3.2 Association between CAR and 28-day mortality in HF patients

Table 2 presents the findings from the multivariable Cox proportional hazards regression analysis. After adjusting for various clinical factors such as age, gender, NYHA classification, and other laboratory markers (Model 3), each 1-unit increase in CAR was associated with a 14% higher risk of mortality within 28 days (HR: 1.14, 95% CI: 1.07–1.21, *P* < 0.001).

**Table 2:** Association of creatinine/albumin ratio and 28-day mortality in heart failure patients.

Exposure	Model 1		Model 2		Model 3	
	HR (95% CI)	*P*-value	HR (95% CI)	*P*-value	HR (95% CI)	*P*-value
CAR	1.15 (1.11∼1.20)	<0.001	1.17 (1.12∼1.23)	<0.001	1.14 (1.07∼1.21)	<0.001

Model 1: no covariates were adjusted.

Model 2: adjusted for age and gender.

Model 3: adjusted for age, gender, NYHA cardiac function classification, platelet, BNP, and potassium.

CAR, creatinine/albumin ratio; NYHA, New York Heart Association; BNP, brain natriuretic peptide; HR, hazard ratio; CI, confidence interval.

### 3.3 Dose-response relationship between CAR and 28-day mortality

**Figure 2:**
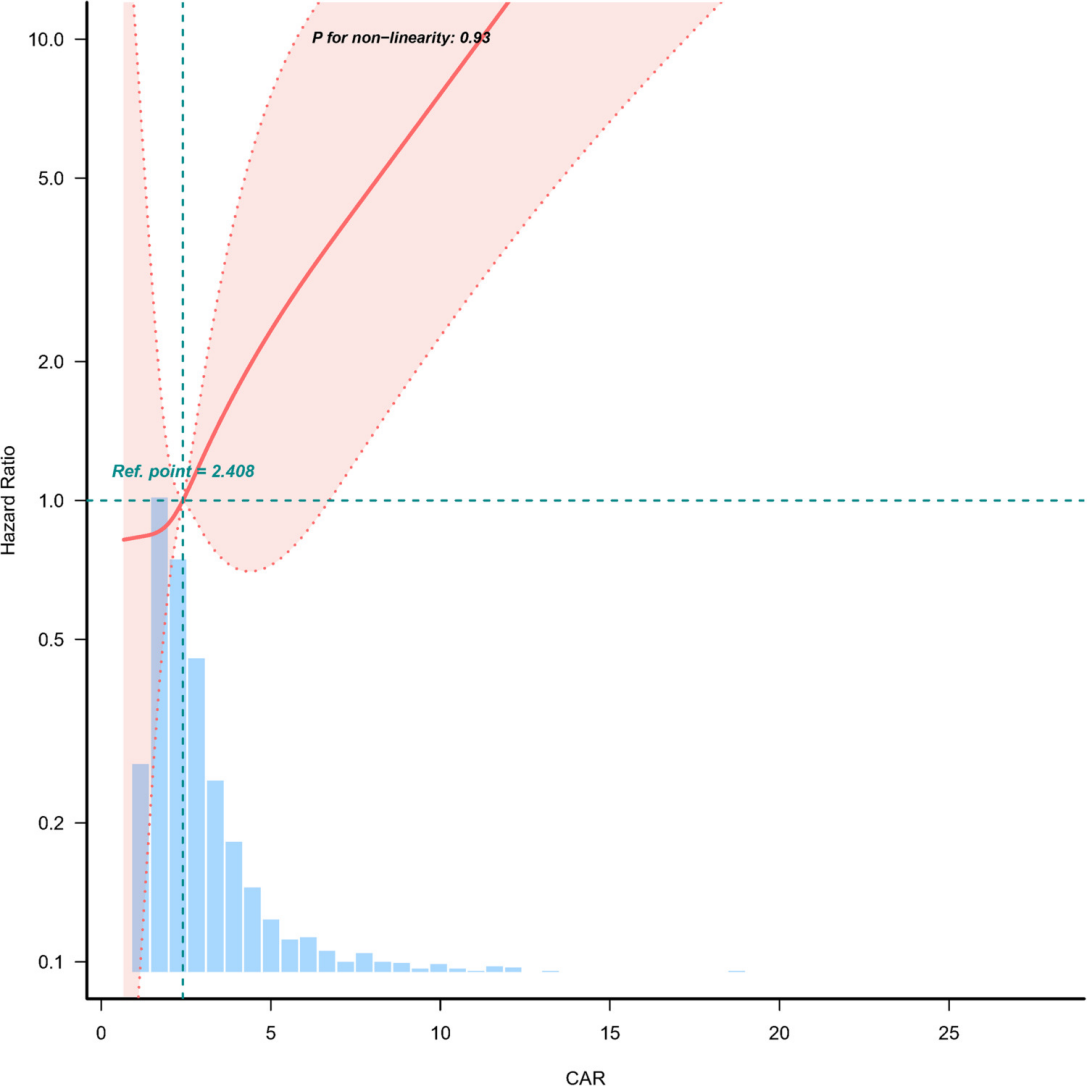
Dose-response relationship between creatinine/albumin ratio and 28-day mortality in heart failure patients. Adjustment factors included age, gender, NYHA cardiac function classification, platelet, BNP, and potassium. The red line and the area between the red dashed lines represents the estimated values and their corresponding 95% confidence intervals, respectively. CAR, creatinine/albumin ratio; NYHA, New York Heart Association; BNP, brain natriuretic peptide; HR, hazard ratio; CI, confidence interval.

A dose-response relationship between CAR and 28-day mortality was observed in Figure 2. The analysis demonstrated a linear association, with an increasing CAR corresponding to a higher risk of 28-day mortality.

### 3.4 Kaplan–Meier survival curve

**Figure 3:**
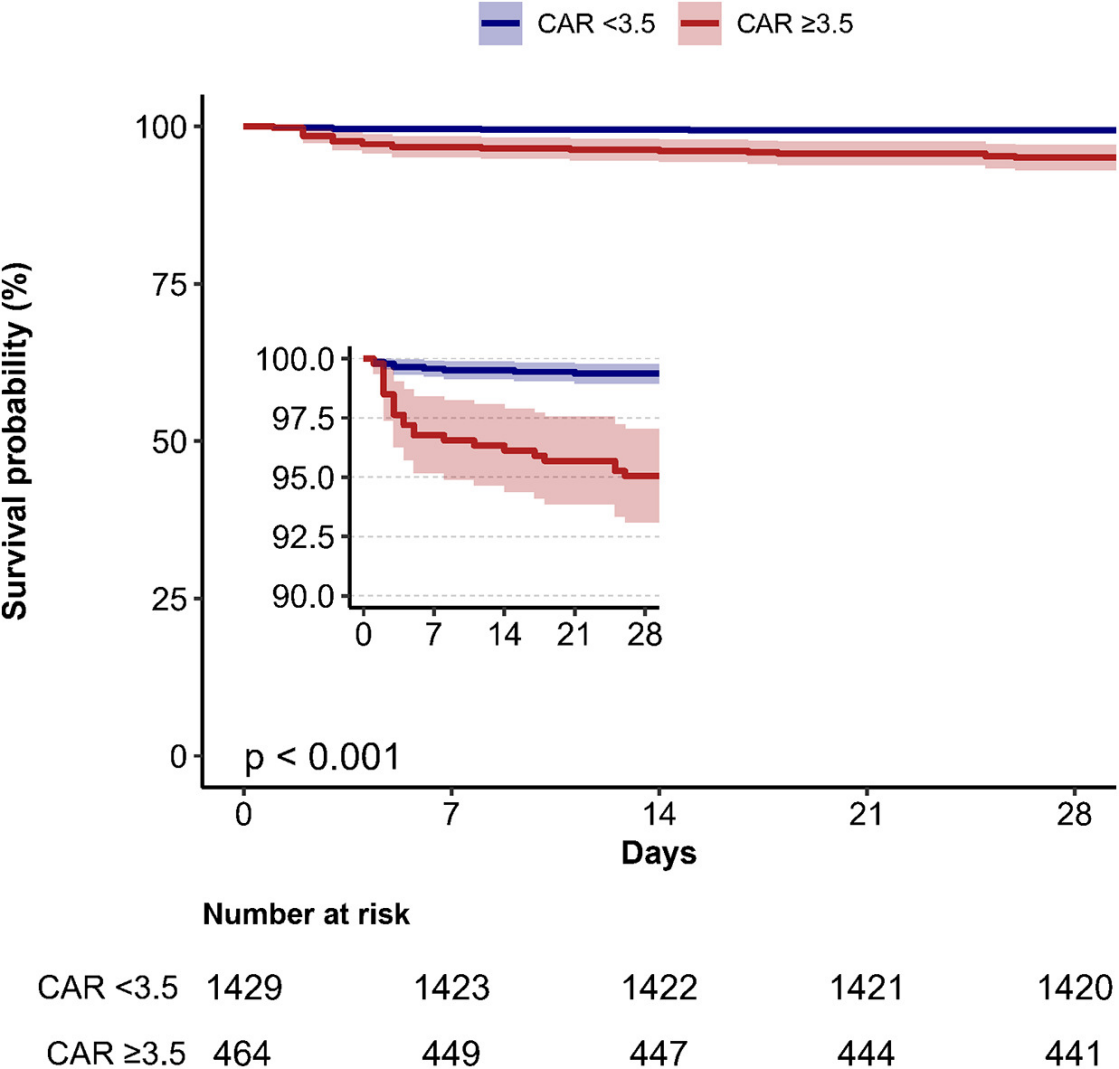
Kaplan–Meier survival curves depicting 28-day mortality stratified by creatinine/albumin ratio categories in heart failure patients. CAR, creatinine/albumin ratio.

Kaplan–Meier survival analysis was conducted to evaluate the 28-day survival probability stratified by CAR categories (Figure 3). Patients with CAR ≥ 3.5 had significantly lower survival rates compared to those with CAR < 3.5 (*P* < 0.001).

### 3.5 ROC curve analysis

**Figure 4:**
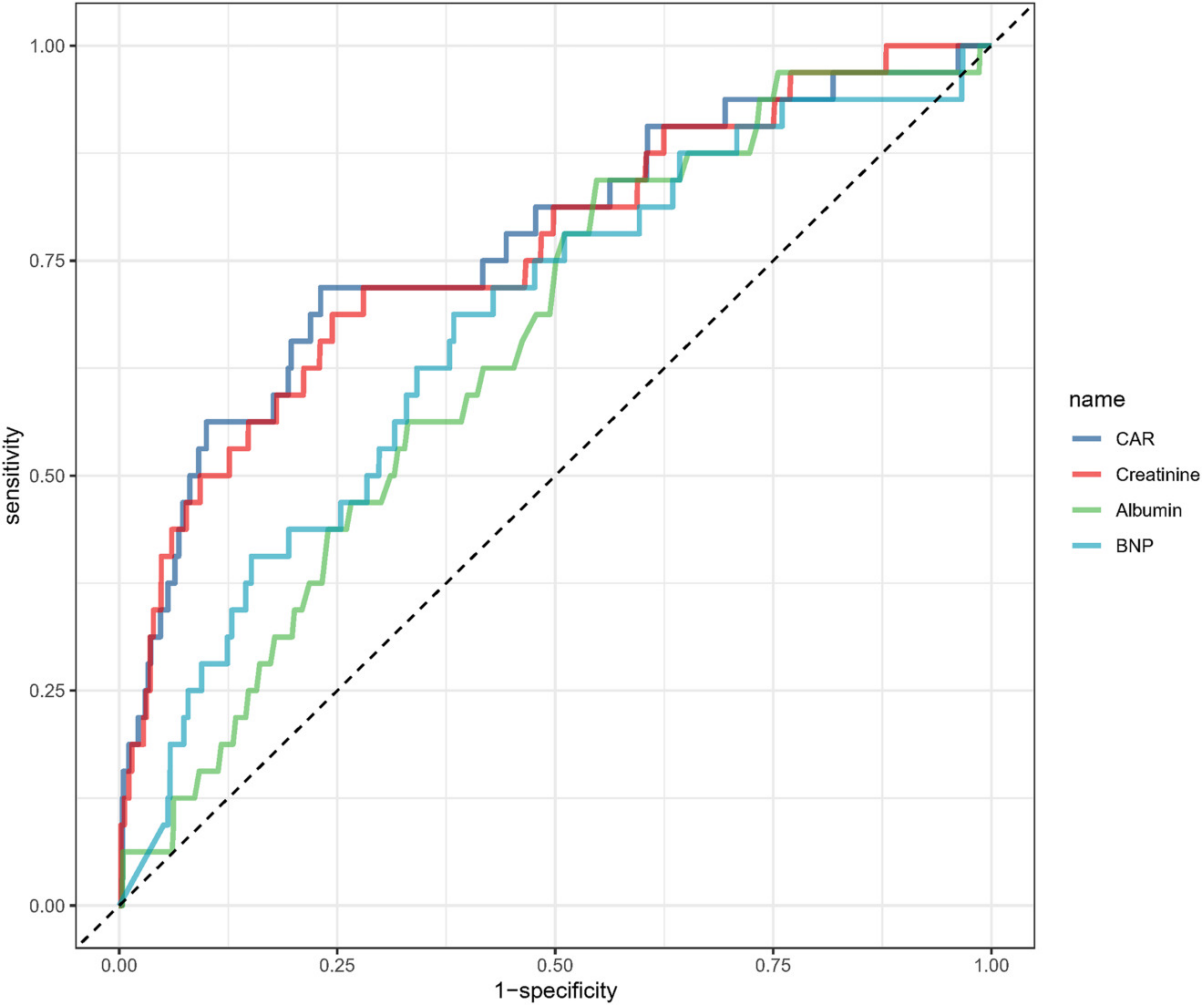
Receiver operating characteristic analysis of creatinine/albumin ratio in predicting 28-day mortality.

The ROC curve analysis was conducted to assess the predictive ability of CAR for 28-day mortality (Figure 4, Table 3). The AUC for CAR was 77.1% (95% CI: 67.6%-86.6%), indicating a good predictive performance. The optimal threshold for CAR was 3.548, with a sensitivity of 0.719 and specificity of 0.769. For comparison, BNP, creatinine and albumin alone had AUCs of 68.0%, 76.2% and 64.9%, respectively, suggesting that the predictive performance of CAR was slightly higher than that of creatinine and markedly better than that of BNP and albumin individually.

**Table 3:** Information of receiver operating characteristic curve in figure 4.

Variables	AUC	95%CI	Threshold	Sensitivity	Specificity
CAR	77.072%	67.591%∼86.554%	3.548	0.719	0.769
Creatinine	76.177%	66.788%∼85.566%	123.05	0.687	0.756
Albumin	64.930%	56.285%∼73.574%	37.35	0.844	0.452
BNP	68.020%	57.457%∼78.583%	1,280.465	0.667	0.654

CAR, creatinine/albumin ratio; BNP, brain natriuretic peptide; AUC, area under the curve; CI, confidence interval.

## 4 Discussion

In this retrospective cohort study, we found that CAR is a significant and independent predictor of 28-day mortality in patients with HF. The relationship between CAR and 28-day mortality was linear, as confirmed by dose-response analysis, and the predictive performance of CAR (AUC: 77.1%) surpassed that of BNP. Subgroup analyses showed consistent associations across most strata. These findings identify the CAR as a prognostic biomarker that simultaneously reflects renal dysfunction and nutritional status, providing critical insights for risk stratification in patients with HF.

The association between CAR and short-term mortality in HF patients remains underexplored, with previous studies primarily focusing on serum creatinine or albumin individually. Elevated serum creatinine has been widely associated with poor outcomes in HF due to its reflection of renal impairment (9, 11, 19), while hypoalbuminemia, indicative of malnutrition and systemic inflammation, has also been linked to higher mortality (10, 12). Additionally, inflammation plays a critical role in HF prognosis, as demonstrated by inflammatory markers such as the C-reactive protein to albumin ratio, which has been associated with long-term mortality in HF with reduced ejection fraction (20). Studies investigating CAR as a combined marker have shown its predictive value in acute coronary syndrome (7) and acute pancreatitis (13). These findings align with our results, further supporting the use of renal and nutritional biomarkers like CAR for risk stratification in HF patients. The relationship between CAR and mortality in HF can be explained by the combined effects of renal dysfunction and malnutrition, both of which are common in HF and contribute to worse outcomes. Renal dysfunction in HF is influenced by multiple interconnected mechanisms (21). Hemodynamic abnormalities, such as increased central venous pressure and reduced cardiac output, significantly impair renal function by altering renal preload and afterload. Sympathetic hyperactivity, characterized by increased renal sympathetic nerve activity, exacerbates sodium retention, reduces renal blood flow, and stimulates renin release. The renin–angiotensin–aldosterone system (RAAS) activation, initially compensatory, leads to long-term adverse effects including fibrosis, oxidative stress, and endothelial dysfunction. Hypoalbuminemia in HF results from a combination of interconnected mechanisms (22). Malnutrition and cardiac cachexia, stemming from reduced appetite, gastrointestinal dysfunction, and increased metabolic demands, diminish hepatic albumin synthesis. Chronic inflammation, marked by elevated cytokines like IL-6 and CRP, further suppresses albumin production and accelerates its breakdown. Hepatic congestion due to elevated venous pressures impairs liver function and albumin synthesis, while severe venous congestion can lead to protein-losing enteropathy, compounding albumin loss. These mechanisms likely interact synergistically, with CAR consolidating their combined impact into a unified predictive biomarker, reflecting a broader spectrum of pathophysiological processes and may provide additional predictive value beyond either marker individually.

This study employed several unique methodological approaches, such as multivariable Cox regression models with progressive adjustments and restricted cubic spline analyses, to elucidate the relationship between CAR and 28-day mortality. Additionally, ROC curve analysis was employed to identify an optimal CAR threshold, providing valuable insights for potential clinical application.

Despite these strengths, several limitations of the study must be acknowledged. First, the retrospective design is inherently prone to biases, including unmeasured confounding and incomplete data, even with the application of multivariate imputation techniques. Second, while the single-center setting ensures consistency in data collection and interpretation, it limits the extrapolation of the findings to broader, more diverse populations with different demographic and clinical characteristics. Third, the analysis was based solely on baseline CAR values, which restricts the ability to assess longitudinal changes in CAR, potentially limiting the understanding of its dynamic prognostic value over time. Fourth, left ventricular ejection fraction and other echocardiographic parameters were unavailable for most patients, preventing stratification by heart failure phenotype and constraining the interpretation of our results.

## 5 Conclusion

In patients with HF, CAR may serve as an independent predictor of 28-day mortality. Its ability to simultaneously reflect renal dysfunction, malnutrition, and inflammation highlights its potential as a valuable biomarker for risk stratification. Further multicenter, prospective studies are needed to confirm its clinical utility and investigate its role alongside other biomarkers in guiding personalized treatment strategies and improving patient outcomes.

## Data Availability

Publicly available datasets were analyzed in this study. This data can be found here: https://physionet.org/content/heart-failure-zigong/1.3/.
