# Supplementary material for: Prognostic value of the serum creatinine/albumin ratio for 28-day mortality in heart failure: a retrospective cohort study
Source: Front Cardiovasc Med. 2025 Jul 9;12:1586327. doi: 10.3389/fcvm.2025.1586327 (PMC12283707; doi:10.3389/fcvm.2025.1586327)
Supplement: Supplementary file 1 [file Table1.doc]

Table S1. Baseline characteristics of participants with missing data compared to those with complete data.

| Covariates | Total | Missing data | Missing case | Complete case | P value |
| --- | --- | --- | --- | --- | --- |
|  | (n = 1893) | (n, %) | (n = 79) | (n = 1814) |  |
| Age, years |  |  |  |  | 0.861 |
| <70 | 511 (27.0) | 0 (0) | 22 (27.8) | 489 (27) |  |
| ≥70 | 1382 (73.0) | 0 (0) | 57 (72.2) | 1325 (73) |  |
| Gender |  |  |  |  | 0.34 |
| Male | 793 (41.9) | 0 (0) | 29 (36.7) | 764 (42.1) |  |
| Female | 1100 (58.1) | 0 (0) | 50 (63.3) | 1050 (57.9) |  |
| BMI, kg/m2 | 20.8 (18.5, 23.4) | 0 (0) | 20.1 (18.6, 23.1) | 20.8 (18.5, 23.4) | 0.285 |
| MAP, mmHg | 94.9 ± 16.2 | 0 (0) | 93.0 ± 15.5 | 95.0 ± 16.2 | 0.291 |
| NYHA cardiac function  classification, n (%) |  |  |  |  | 0.216 |
| II | 331 (17.5) | 0 (0) | 18 (22.8) | 313 (17.3) |  |
| III | 983 (51.9) | 0 (0) | 43 (54.4) | 940 (51.8) |  |
| IV | 579 (30.6) | 0 (0) | 18 (22.8) | 561 (30.9) |  |
| Myocardial infarction, n (%) | 136 ( 7.2) | 0 (0) | 5 (6.3) | 131 (7.2) | 0.764 |
| Cerebrovascular disease, n (%) | 142 ( 7.5) | 0 (0) | 7 (8.9) | 135 (7.4) | 0.639 |
| COPD, n (%) | 224 (11.8) | 0 (0) | 8 (10.1) | 216 (11.9) | 0.631 |
| Diabetes, n (%) | 439 (23.2) | 0 (0) | 13 (16.5) | 426 (23.5) | 0.147 |
| Solid tumor, n (%) | 36 ( 1.9) | 0 (0) | 2 (2.5) | 34 (1.9) | 0.660 |
| WBC, 10^9^/L | 7.3 ± 3.5 | 16 (0.8) | 7.2 ± 4.3 | 7.3 ± 3.5 | 0.698 |
| Hemoglobin, g/L | 114.9 ± 24.3 | 17 (0.9) | 110.6 ± 25.2 | 115.0 ± 24.3 | 0.160 |
| Platelet, 10^9^/L | 144.5 ± 64.2 | 16 (0.8) | 145.6 ± 60.9 | 144.5 ± 64.3 | 0.890 |
| Hs‐cTn, pg/mL | 0.1 (0.0, 0.1) | 55 (2.9) | 0.1 (0.0, 0.1) | 0.1 (0.0, 0.1) | 0.936 |
| BNP, pg/mL | 768.1 (319.2, 1762.3) | 22 (1.2) | 525.8 (233.8, 1127.2) | 775.4 (326.8, 1784.9) | 0.059 |
| Potassium, mmol/L | 4.0 ± 0.7 | 3 (0.2) | 4.1 ± 1.0 | 4.0 ± 0.7 | 0.253 |
| Sodium, mmol/L | 138.3 ± 4.9 | 3 (0.2) | 137.7 ± 7.1 | 138.3 ± 4.8 | 0.275 |
| Serum creatinine, μmol/L | 87.4 (65.0, 123.7) | 0 (0) | 86.2 (63.0, 121.1) | 87.6 (65.1, 123.8) | 0.585 |
| Albumin, g/L | 36.5 ± 5.0 | 0 (0) | 35.6 ± 5.0 | 36.6 ± 5.0 | 0.104 |
| CAR | 2.4 (1.8, 3.5) | 0 (0) | 2.3 (1.7, 3.4) | 2.4 (1.8, 3.5) | 0.926 |
| Mortality, n (%) | 32 ( 1.7) | 0 (0) | 0 (0) | 32 (1.8) | 0.642 |

Abbreviation: BMI, body mass index; MAP, mean arterial pressure; NYHA, New York Heart Association; COPD, chronic obstructive pulmonary disease; WBC, white blood cell; hs‐cTn, high sensitivity cardiac troponin; BNP, brain natriuretic peptide; CAR, creatinine/albumin ratio.
