# Supplementary material for: Prognostic value of the serum creatinine/albumin ratio for 28-day mortality in heart failure: a retrospective cohort study
Source: Front Cardiovasc Med. 2025 Jul 9;12:1586327. doi: 10.3389/fcvm.2025.1586327 (PMC12283707; doi:10.3389/fcvm.2025.1586327)
Supplement: Supplementary file 2 [file Table2.doc]

Table S2. Comparison of Multivariable Cox Models Based on Multiple Imputation and Complete-Case Analysis

|  | Exposure | | Model 1 | | Model 2 | | Model 3 | |
| --- | --- | --- | --- | --- | --- | --- | --- | --- |
|  |  | | HR (95% CI) | P value | HR (95% CI) | P value | HR (95% CI) | P value |
| Imputed Dataset | | CAR | 1.15 (1.11~1.20) | <0.001 | 1.17 (1.12~1.23) | <0.001 | 1.14 (1.07~1.21) | <0.001 |
| Complete-Case Dataset | | CAR | 1.15 (1.11~1.20) | <0.001 | 1.17 (1.12~1.23) | <0.001 | 1.13 (1.07~1.21) | <0.001 |

Model 1: no covariates were adjusted.

Model 2: adjusted for age and gender.

Model 3: adjusted for age, gender, NYHA cardiac function classification, platelet, BNP, and potassium.

Abbreviation: CAR, creatinine/albumin ratio; NYHA, New York Heart Association; BNP, brain natriuretic peptide; HR, hazard ratio; CI, confidence interval.
